# MicroRNA-21 Increases Proliferation and Cisplatin Sensitivity of Osteosarcoma-Derived Cells

**DOI:** 10.1371/journal.pone.0161023

**Published:** 2016-08-11

**Authors:** Vanita Vanas, Barbara Haigl, Verena Stockhammer, Hedwig Sutterlüty-Fall

**Affiliations:** 1 Institute of Cancer Research, Department of Medicine I, Medical University of Vienna, Vienna, Austria; 2 Department of Orthopaedics, Medical University of Vienna, Vienna, Austria; University of Navarra, SPAIN

## Abstract

Osteosarcoma is the most common primary bone tumor and poor prognosis for osteosarcoma patients is mainly due to chemotherapy resistance. MicroRNAs are important to maintain pathophysiological mechanisms of cancer and influence cell sensitivity to chemotherapy. In this study, we tested the functions of microRNA-21 for malignant features as well as for drug resistance of osteosarcoma. We used Northern blot to measure microRNA-21 levels in osteosarcoma-derived cell lines. MicroRNA-21 activity was modulated by either expressing a sponge to decrease its activity in an osteosarcoma-derived cell line expressing high levels of microRNA-21 or by introducing pri-microRNA-21 in a cell line with low endogenous levels. Cell migration was determined in a scratch assay and cell proliferation was measured by performing growth curve analysis. Sensitivity of the cells towards chemotherapeutics was investigated by performing cell viability assays and calculating the IC50 values. While cell migration was unaffected by modulated microRNA-21 levels, microRNA-21 inhibition slowed proliferation and exogenously expressed microRNA-21 promoted this process. Modulated microRNA-21 activity failed to effect sensitivity of osteosarcoma-derived cell lines to doxorubicin or methotrexate. Contrarily, reduction of microRNA-21 activity resulted in enhanced resistance towards cisplatin while ectopic expression of microRNA-21 showed the opposite effect. Increased microRNA-21 levels repressed the expression of Sprouty2 and ectopic expression of Sprouty2 was able to largely rescue the observed effects of microRNA-21 in osteosarcoma. In summary, our data indicate that in osteosarcoma microRNA-21 expression is an important component for regulation of cell proliferation and for determining sensitivity to cisplatin.

## Introduction

Osteosarcoma are the most common malignant bone disease primarily localized at the long bones and characterized by a high propensity for metastasis, especially to the lung [1]. Due to neoadjuvant and post-surgery chemotherapy significant survival gains were made from the 1960s to the 1980s, but since then patient’s survival rate leveled [2]. To improve patients’ treatment further, new insights into processes involved in tumorigenesis and therapeutic resistance are urgently needed.

MicroRNAs (miRNAs or miRs) are 18 to 25 nucleotides (nt) long, endogenously expressed, noncoding RNAs with important biological functions. MiRNAs are processed in the nucleus from RNA polymerase II produced primary transcripts (pri-miRNA) to 70 nt long precursor miRNA (pre-miR). In the cytoplasm, pre-miR is cleaved and the mature, single stranded miR is incorporated into a ribonucleotide protein complex which functions as a miRNA-induced silencing complex. Usually, miRNAs facilitate degradation of target mRNA or inhibit their translation [3, 4]. Changes in the miRNA profiles are characteristic for a variety of tumors. Many miRNAs including miR-21 are able to function as oncogenes (oncomirs) or as tumor suppressors [5].

MiR-21 was found to be up-regulated in nearly all solid tumors, including osteosarcoma [6], lung [7, 8], colorectal [9, 10], breast [11, 12], liver [13] as well as head and neck cancer [14]. Many miR-21 targets code for tumor suppressors, with a role in inhibiting cell signaling, cell proliferation and migration, e.g. phosphatase and tensin homolog (PTEN) tumor suppressor [13], Sprouty1 (Spry1) [15] and Sprouty2 [16]. Additionally, miR-21 influences modulators of cell division, such as Cell division cycle 25 homolog A [17] or apoptosis like Programmed Cell Death 4 Protein (PDCD4) [18, 19]. The metastatic process can also be influenced by miR-21 via regulation of substrates like Topomyosin [20], Reck und TIMP3 [21], factors which are known to modulate the extracellular matrix via metalloproteases.

In an earlier study in osteosarcoma-derived cells, we observed that Spry2 could function as a tumor suppressor, while Spry4 had no influence on the malignant phenotype of the cells [22]. The expression of both Spry proteins is induced by mitogen-induced signaling [23], but in contrast to Spry4, Spry2 is additionally shown to be modulated by miR-21 [16]. Therefore, in the presented study, we investigated the influence of modulated miR-21 expression on the malignant phenotype of osteosarcoma-derived cell lines. In addition, we analyzed if miR-21 levels impact susceptibility of the cells to the standard chemotherapy regimens in osteosarcoma treatment.

## Materials and Methods

### Plasmid constructs

As a first step the luciferase sequence was transferred from the pGL3 (Promega) into the pAdlox plasmid using *HindIII/XbaI* sites. The pBabepuro construct expressing luciferase (pBluc) was then generated by cloning the luciferase sequence (from pAdlox luciferase vector) into *BamHI/EcoRI* digested pBabepuro vector (pBp). The miR-21 sponge was produced by consecutive introduction of oligonucleotides. In a first step two oligonucleotides 5’-GAT CCT CAA CAT CAG TCT GAT AAG CTA CCT CGA GTC AAC ATC AGT CTG ATA AGC TAG-3’ and 5’-AAT TCT AGC TTA TCA GAC TGA TGT TGA CTC GAG GTA GCT TAT CAG ACT GAT GTT GAG-3’ were phosphorylated, annealed and ligated into the *EcoRI/BamHI* digested pAdlox luciferase vector. This intermediate product was cleaved with *XbaI/BamHI* and in two consecutive steps oligonucleotides harboring again two binding sites 5’- CTA GAG ATC GGA TCC TCA ACA CAG TCT GAT AAG CTA CCT CGA GTC AAC ATC AGT CTG ATA AGC TAC-3’ and 5’-GAT CGT AGC TTA TCA GAC TGA TGT TGA CTC GAG GTA GCT TAT CAG ACT GAT GTT GAG GAT CCG ATCT-3’ were annealed and cloned 3’ to the luciferase coding sequence. Via an intermediate cloning procedure cloning the *HindIII/BglI* fragment to the *HindIII/BamHI* sites of the pGL3 basic vector, the sponge sequence was transferred into a *BamHI/EcoRI* digested pBp vector.

For ectopic expression of miR-21, a 979 base pair (bp) fragment of primiR-21 including the sequence of premiR-21 was amplified via PCR using oligonucleotides 5’-CTA ATC CAC CTA CAA CAA GA-3’ as forward primer and 5’-GAT ACT TCT AGA TTT TCA AAG AAG GTC AAG TA-3’ reverse primer. The PCR fragment was ligated into the *BamHI/EcoRI* sites of pBp plasmid using the described subcloning steps via pAdlox and pGL3 vector.

All constructs were verified by sequencing (Microsynth).

### Cell culture

Osteosarcoma-derived human cell lines U2OS, MG63, 143B, HOS and SaOS2 were purchased from American Type Culture Collection. HLNG cell line was established at the Institute of Cancer Research Vienna [22]. All cell lines were cultured at 37°C in 7.5% CO_2_ using recommended medium supplemented with 10% fetal calf serum (FCS), penicillin (100 U/ml) and streptomycin (100 μg/ml).

### Adenoviral infection

Adenoviruses were prepared earlier [22]. One million cells were seeded on a 10cm diameter plate and infected with a multiplicity of infection of 25.

### Cell transfection and selection

CaPO_4_ transfection was carried out as specified [24]. Media supplemented with 2μg/ml puromycin selected for successfully transfected cells. To generate mixed clones (MC) expressing the sponge, after a week of selection, cell plates with more than 100 clones were trypsinized to combine the cells from all clones. For analyzing miR-21 overexpression 10 single clones were isolated and propagated.

### miRNA Northern blot

RNA isolation and miRNA Northern blotting were performed as described previously [25]. Probe for Homo sapiens U6 small nuclear 2 RNA (U6) was used to serve as loading control.

### Immunoblot

Protein isolation and immunoblotting were carried out as described [26]. Spry-specific antibodies were produced earlier [23, 26, 27] as described [27]. Antibodies recognizing PDCD4 (monoclonal, rabbit antibody EPR3431 from Epitomics was diluted 1:5000) and PTEN (monoclonal rabbit antibody EPR4408 from Epitomics was diluted 1:1000) were purchased. As loading control antibodies against beta-actin (monoclonal, mouse antibody AC-15 from Novus Biological was diluted 1:10 000) and GAPDH (monoclonal, mouse antibody G-9 from Santa Cruz was diluted 1: 30 000) were used.

### Luciferase assay

6.5x10^4^ logarithmically growing cells were harvested and luciferase assays were performed according to Dorriguzzi et al [28].

### Growth curves

To investigate the influence of miR-21 modulation on cell proliferation, 1-2x10^4^ cells were seeded into 6cm diameter dishes and counted every 24 hours. The growth curves were performed and doubling times were calculated [22].

### Scratch assay

Migration velocity was determined by using a scratch assay [29]. Cell images were taken at 1, 3, 5, 8, 10 and 12 hours at four to five different focal areas.

### Cell viability assay

To analyze cell viability, 1x10^5^ cells were seeded in 6cm diameter dishes in duplicates. After 24 hours, cisplatin (Sigma Aldrich), doxorubicin hydrochloride (Sigma Aldrich) or methotrexate hydrate (Sigma Aldrich) was added to the cells in three different concentrations. 48 hours later, cells were counted using a Neubauer counting chamber. Relative cell number was obtained by setting corresponding untreated controls as 1. IC50 values were calculated using Graph Pad Prism 5 software by applying Point-to-Point function.

## Results

### Reduced miR-21 activity causes decelerated proliferation of osteosarcoma-derived cells

**Fig 1:**
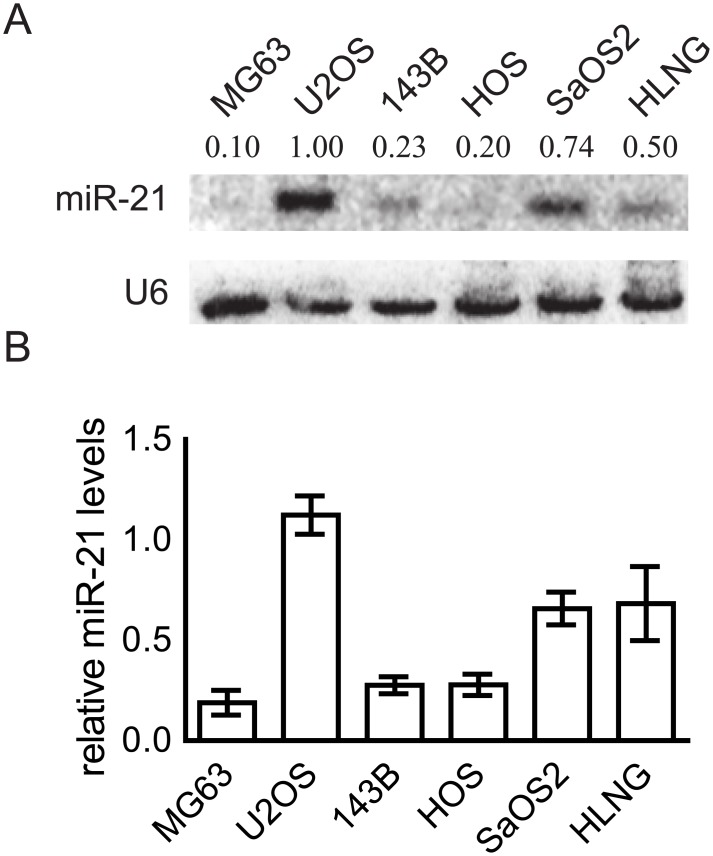
Endogenous expression of miR-21 in osteosarcoma-derived cell lines. Logarithmically growing cells were harvested to perform a miRNA Northern blot. (A) One representative Northern blot measuring miR-21 levels of six osteosarcoma-derived cell lines is shown. U6 served as loading control. (B) Results of two to three Northern blots were quantified using Image Quant 5.0. The miR-21/U6 ratios were determined and depicted as a block diagram. Means ± SEM are shown.

As an initial experiment, the miR-21 expression levels in six logarithmically growing osteosarcoma-derived cell lines were determined. All cell lines express miR-21, but the levels differ significantly (Fig 1A). While in U2OS, SaOS2 and HLNG miR-21 expression is high, in the other three cell lines (MG63, 143B and HOS cells) the levels are about five fold lowered compared to U2OS showing the highest miR-21 expression (Fig 1B). Due to this data, we selected U2OS cells to perform the experiments with inhibited miR-21 activity.

**Fig 2:**
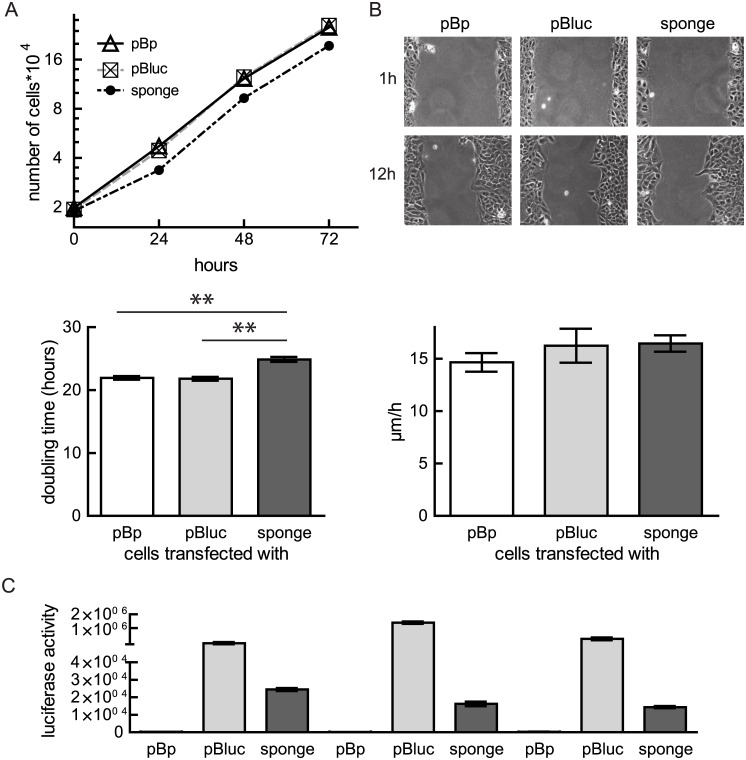
Influence of reduced miR-21 activity on cell proliferation and migration. U2OS were transfected with either pBabepuro (pBp), pBabepuro luciferase (pBluc) or a miR-21 sponge. (A) Cell proliferation was measured by counting the cells daily. A representative growth curve is depicted (Means ± SEM). Using exponential growth equation, doubling times were calculated using Graph Pad Prism 5 (Means ± SEM). Values from six experiments of three clones are depicted and significance was calculated. **p < 0.01 (B) A scratch assay with U2OS cells transfected with the indicated plasmids was performed. Representative pictures of cells 1 and 12 hours after scratch was set are shown (upper panel). Migration velocity was calculated using linear regression. Means ± SEM of three independent experiments are shown (lower panel). (C) Transfection efficiency was verified by measuring luciferase activity of the selected mixed clones (MC1-3) (Means ± SEM).

Activity of miRNAs can be effectively regulated by so-called “sponges”, ectopically or endogenously produced competitive RNAs, which buffer the activity of miRNAs by sequestering them from their natural targets. In order to antagonize the endogenous miR-21 activity, we cloned consecutive arranged binding sites (n = 6) for miR-21 3’ to the luciferase coding sequence and used this sponge to study the influence of reduced miR-21 amounts on the malignant phenotype of osteosarcoma cells. U2OS cells (with high miR-21 levels) were transfected with miR-21 sponges and puromycin-selected for a week. Proliferation was measured by performing growth curves of mixed cell populations. While U2OS cells selected for the empty vector (pBp) (21.95 ± 0.3 hours) or the luciferase expressing vector (21.88 ± 0.3 hours) double in less than 22 hours, expression of a miR-21 sponge decelerates cell proliferation to less than one doubling per day (24.88 ± 0.4 hours) (Fig 2A). In parallel, we tested how expression of the miR-21 sponges influences cell migration of the established mixed clones (Fig 2B) by performing a scratch assay. In both, control treated cells as well as in the sponge expressing U2OS cells at 12 hours the closure of the scratch is obviously comparable progressed. Independent of the transfected vectors all U2OS populations migrate with a velocity of about 15 μm/hour (Fig 2B).

Measurement of luciferase activities verified the effectiveness of the transfection and selection process (Fig 2C). The strongly reduced activity in the mixed cell clones derived from transfection of the sponges indicates that endogenous miR-21 is titrated by the miR-21 binding sites (Fig 2C).

Taken together, our data demonstrate that reduced miR-21 activity interferes with proliferation of U2OS cells.

### Inhibition of miR-21 activity desensitizes U2OS cells to cisplatin treatment

**Fig 3:**
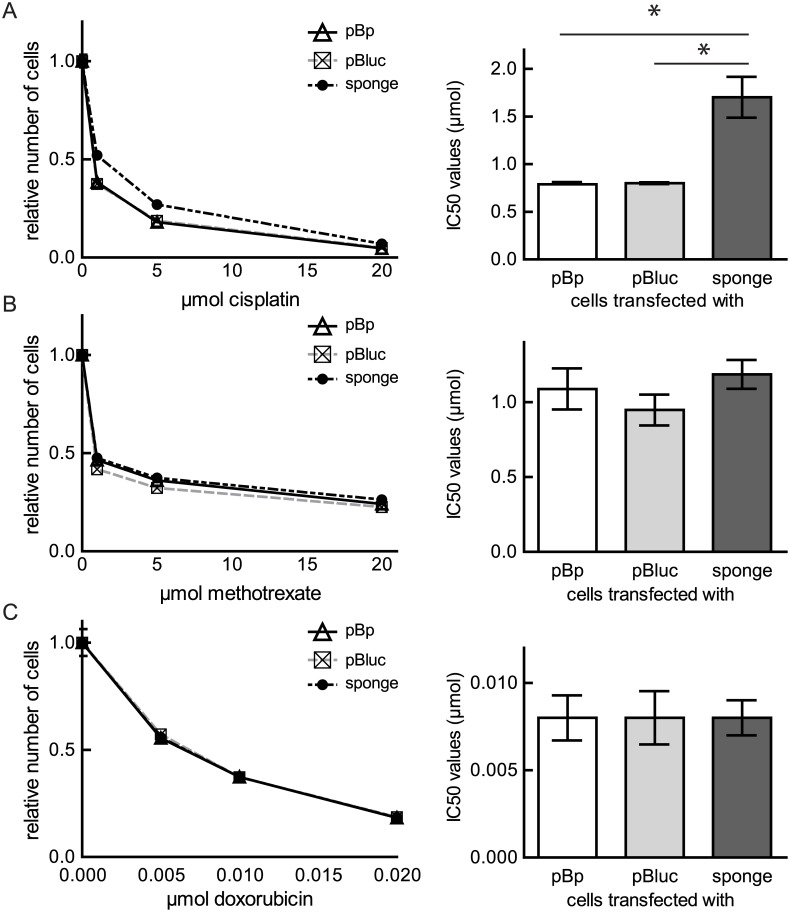
Influence of reduced miR-21 activity on the sensitivity of U2OS cells towards chemotherapeutic drugs. Logarithmically growing MCs were incubated for 48 hours to the indicated increasing concentration of cisplatin (A), methotrexate (B) or doxorubicin (C) and a cell viability assay was performed (left panel). Means ± SEM of a representative experiment are presented as curves. The IC50 values of three to four experiments are depicted as means ± SEM and an unpaired t-test was performed; *p < 0.05 (right panel).

As treatment for osteosarcoma a combination of cisplatin with doxorubicin, and methotrexate is frequently used. Genetic differences in the tumors influence the response to chemotherapeutic agents and cause inter-individual differences in treatment outcomes [30]. Therefore, we assessed whether miR-21 activity is a relevant factor for osteosarcoma in conventional chemotherapy. We used the mixed clones established after transfection of either pBp, pBluc or the sponge. U2OS cells were sensitive to cisplatin at 48 hours. Treatment with increasing concentrations of cisplatin (1 μmol, 5 μmol, 20 μmol) caused a dose dependent decrease in the number of viable cells. Inhibition of miR-21 activity by the sponge decreased the sensitivity of U2OS to cisplatin and resulted in a significant augmentation of the calculated IC50 value (Fig 3A). Furthermore, U2OS cells expressing the miR-21 sponge or the respective controls were exposed to different concentrations of methotrexate (Fig 3B) and doxorubicin (Fig 3C). Both compounds inhibited cell proliferation and/or viability in a dose-dependent manner. Reduction of miR-21 activity resulted in no significant changes in growth at 48 hours (Fig 3B and 3C). These results suggest that miR-21 activity is a factor in determining the response to cisplatin treatment in osteosarcoma.

### Ectopic expression of miR-21 accelerates cell proliferation and sensitizes osteosarcoma cells towards cisplatin treatment

**Fig 4:**
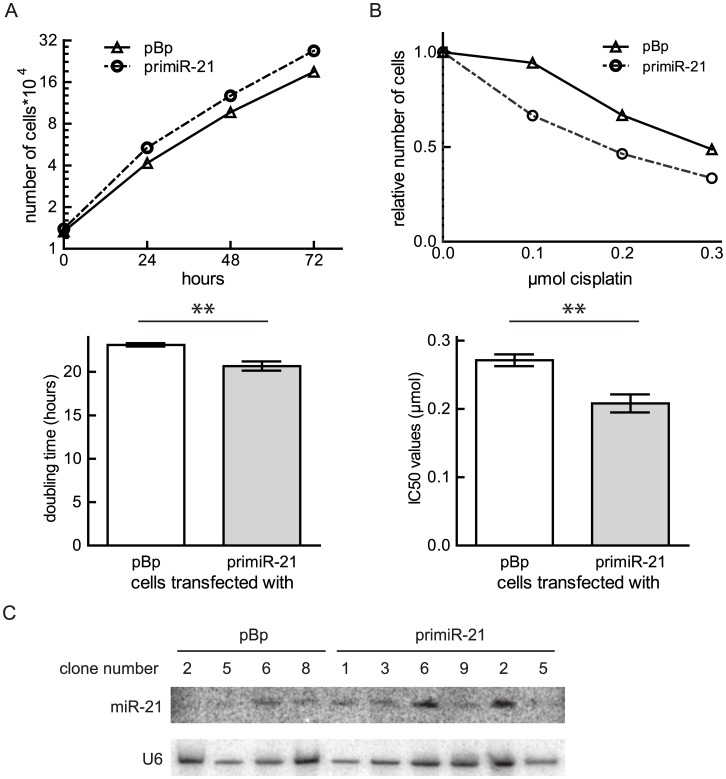
Influence of ectopic miR-21 expression on cell proliferation and cisplatin sensitivity of MG63 cells. Cells were transfected with pBp or primiR-21 and single clones were selected. (A) A representative growth curve of one clone is depicted as means ± SEM. Doubling times of six experiments with three different clones were calculated, the means ± SEM are presented in a column graph and analyzed using unpaired t-test. **p < 0.01. (B) A cell viability assay of a representative single clone obtained after transfection with the indicated plasmid is presented as means ± SEM. IC50 values of three different clones were calculated. Means ± SEM summarize the data. The significance was determined using an unpaired t-test. **p < 0.01 (C) MiRNA Northern blots of the indicated clones obtained after transfection with primiR-21or pBp are shown.

To verify the role of miR-21 for proliferative capacities of osteosarcoma, we additionally investigated if increased miR-21 levels can influence cell proliferation of a cell line with low endogenous miR-21 expression. Therefore, a vector expressing the primiR-21 was transfected into MG63 cells before cells were selected on puromycin. Ten clones were isolated and three clones (1, 2 and 6) overexpressing miR-21 (Fig 4C) were chosen to perform growth curve analyses. The primiR-21 transfected cells clearly accelerated cell proliferation. Cells overexpressing miR-21 divide in about 20 hours, while control treated cells need on average 23.12 ± 0.2 hours (Fig 4A). Next, we asked whether in MG63 cells modulated miR-21 levels as well influenced the sensitivity to cisplatin. Hence, the clones expressing increased levels of miR-21 and their respective controls were exposed to *in vitro* treatment with cisplatin. In comparison to U2OS, MG63 cells were more sensitive to cisplatin treatment and 0.1 μmol, 0.2 μmol, and 0.3 μmol of the agent were used. The cell number was determined 48 hours after drug addition, and IC50 values were calculated. The results showed a decrease in the cell viability in response to cisplatin treatment if miR-21 expression was elevated (Fig 4B). These data demonstrate that independent of the cell line miR-21 promotes cell proliferation of osteosarcoma and verify that miR-21 expression sensitizes osteosarcoma cells to cisplatin treatment.

### Osteosarcoma-derived cells with ectopically augmented miR-21 levels show lowered expression of Spry2 and Spry1

**Fig 5:**
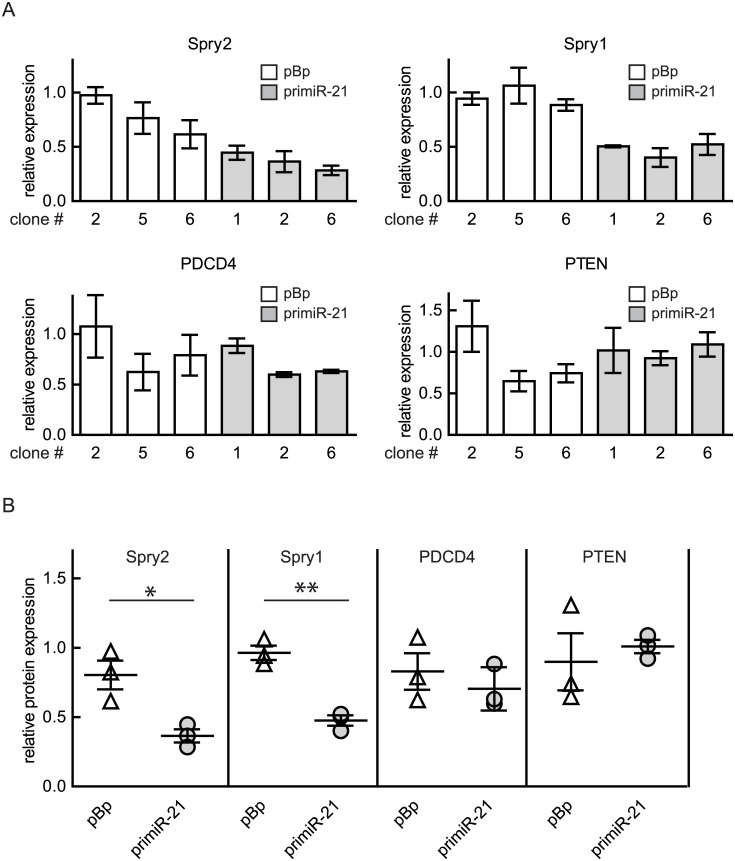
Influence of ectopic miR-21 expression on potential target proteins. Protein lysates of cell clones obtained after transfection and selection of MG63 cells with either pBp or primiR-21 were analyzed using an immunoblot. Protein expression was calculated using Image quant 5.0 software. The highest value was arbitrarily set as 1. GAPDH was used as reference value. (A) Means ± SEM of two to three independent experiments are depicted. The analyzed protein is indicated as title. (B) Calculated levels of the indicated proteins in the clones overexpressing miR-21 were compared to the one in the corresponding control clones. Means ± SEM are presented. Significance was calculated using unpaired t-test. *p < 0.05; **p < 0.01.

In order to analyze the influence of increased miR-21 expression on potential target mRNAs, the levels of some proteins reported to be influenced by miR-21 (Spry2, Spry1, PDCD4 and PTEN) were compared in the primiR-21 expressing and the respective control MG63 clones. As summarized in Fig 5, Spry2 and Spry1 protein levels were lowered in all three primiR-21 expressing clones when compared to the vector transfected cells. Interestingly, the levels of PDCD4 and PTEN, which could function as miR-21 substrates, were comparable in the investigated cell clones (Fig 5A and 5B).

These observations indicate that elevated miR-21 expression in the osteosarcoma-derived cell line MG63 causes a repression of Spry2 and Spry1 protein levels.

### Ectopic Spry2 expression interferes with the miR-21-induced effect on cell proliferation

**Fig 6:**
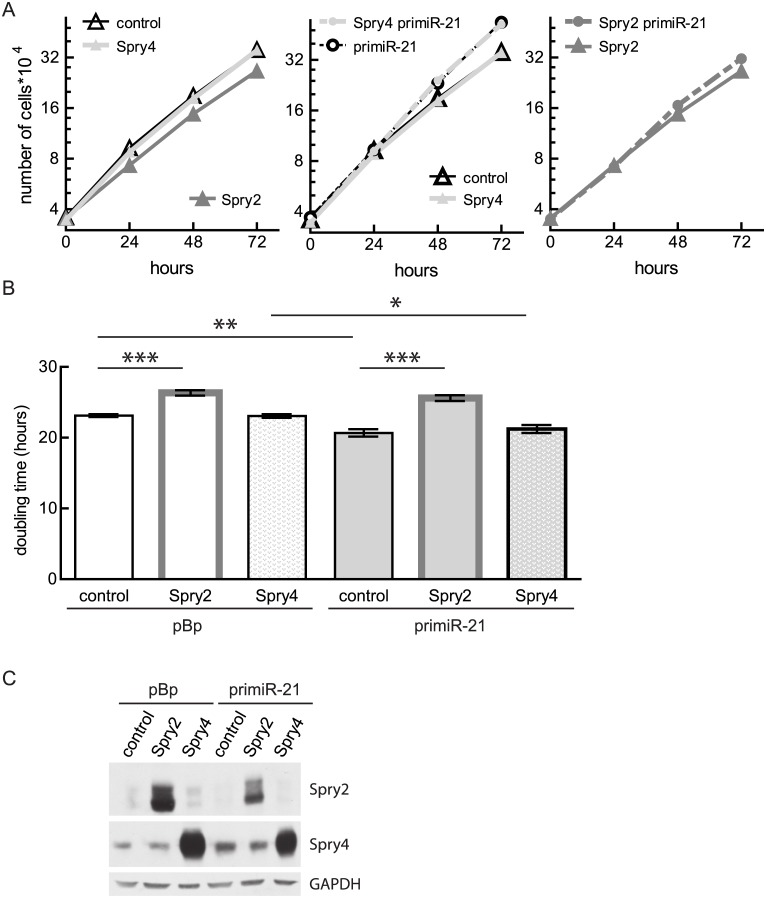
Influence of Spry2 and Spry4 expression on proliferation of miR-21 overexpressing cells. Prior to growth curve analysis, MG63 empty vector or primiR-21 expressing clones were infected with either an adenovirus expressing lacZ (control), Spry2 or Spry4. (A) Representative curves depicting means ± SEM are presented. The ectopically expressed genes are indicated as legend. (B) Doubling times of three to six independent experiments were calculated and depicted as means ± SEM. Significance was determined by unpaired t-test. *p < 0.05; **p < 0.01; ***p < 0.001 (C) Protein lysates of the infected clones were analyzed by performing an immunoblot with the indicated antibodies.

In an earlier study, we observed that Spry2 inhibits cell proliferation of osteosarcoma-derived cell lines [22]. To study if the observed decrease in Spry2 levels is important for the influence of miR-21 on cell proliferation, we performed growth curves with MG63 clones infected with adenoviruses expressing either Spry2 or lacZ (control). The virus-introduced Spry2 mRNA lacks the miR-21 regulatory elements. As a further control, we included cells expressing Spry4, another member of the Spry family which lacks a miR-21 target sequence. In agreement with earlier analysis [22], Spry2 expression inhibited cell proliferation of the control MG63 cells while Spry4 had no influence (Fig 6A left panel and Fig 6B). Furthermore, Spry4 expression had no impact on the accelerating effect of increased miR-21 expression on cell proliferation (Fig 6A middle panel and Fig 6B). If MG63 cells ectopically express Spry2, the augmented miR-21 levels were irrelevant for the cell doubling times (Fig 6A right panel and Fig 6B). The expression of the ectopically introduced Spry proteins was verified by immunoblots (Fig 6C). These data indicate that targeting Spry2 is important for the miR-21-mediated effects on cell proliferation of osteosarcoma.

### Spry2 expression rescues the miR-21 mediated influence on cisplatin-sensitivity

**Fig 7:**
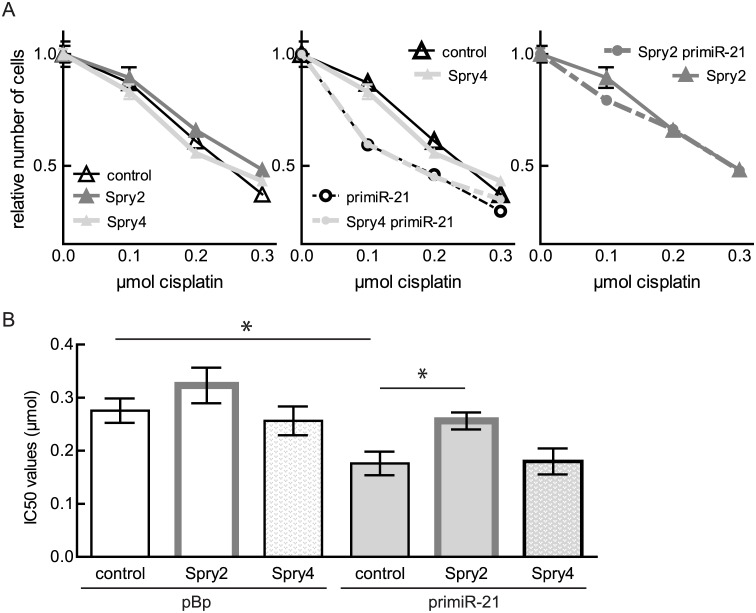
Influence of ectopic Spry protein expression on cisplatin-sensitivity of MG63 cells with different miR-21 expression levels. MG63 clones expressing an empty vector (pBp) or a primiR-21 were infected with adenoviruses expressing the indicated proteins. LacZ was used as control protein. (A) The cell viability assays of representative clones infected with the indicated proteins (see legend) are shown as curves (Means ± SEM). (B) IC50 values of three different clones are summarized as means ± SEM and a t-test was performed. *p < 0.05.

The cell viability assays in the presence of increased amounts of cisplatin revealed that neither Spry2 nor Spry4 expression were significantly influencing the sensitivity of MG63 cells towards this compound (Fig 7A left panel and Fig 7B). The observed increase of the cisplatin-sensitivity in case of augmented miR-21 expression was not affected by Spry4 expression (Fig 7A middle panel and Fig 7B). In contrast, ectopical expression of Spry2 could at least partially abandon the influence of miR-21 on chemosensitivity (Fig 7A right panel and Fig 7B). These data demonstrate that targeting Spry2 expression is involved in the cisplatin-sensitizing effect of miR-21.

## Discussion

MiRNAs are shown to play a substantial role in the pathogenesis of human cancer. In many tumor entities, diagnostic and prognostic miRNA expression profiles can be generated and many miRNAs can function as oncogenes indicating that miRNAs could serve as novel targets for cancer treatment. This could have important implications for diagnosis and treatment of these cancers. With stagnating survival rates in osteosarcoma, the most frequent primary malignant bone tumor, new treatment options are needed and miRNAs may contribute to the development of advancements in osteosarcoma therapy serving as targets or as biomarkers [31]. MiR-21 is one of the miRNAs which has been classified as an oncomir. The expression has been found to be deregulated in almost all types of cancers [32]. Also in osteosarcoma an increase of this oncomir was found in the serum [33] as well as in the tissue [6] and in patient-derived cell lines [34]. This augmentation in the malignant tissue implies a role for this regulatory miRNA in the initiation and progression of this cancer.

In the current study, we analyzed the function of miR-21 in osteosarcoma-derived cell lines. We showed that its expression levels differ strongly in the different cell lines. In accordance with a proposed oncogenic function, miR-21 was shown to perform a function in facilitating invasion capacities of osteosarcoma-derived cells [6, 35]. In this report, we demonstrated for the first time that reduction of miR-21 activity inhibites cell proliferation of osteosarcoma-derived cells while increasing miR-21 expression accelerates their doubling. A stimulating influence of miR-21 on cell proliferation was also described for cells originating from other tumor entities like NSCLC [36], colon [37] or skin [38]. Interestingly, we found migration to be not influenced by modulated miR-21 activity. This is in contrast to reports analyzing influence of miR-21 in breast cancer-derived cell lines [39], in glioma [21] and in liver cancer [13]. In accordance with our findings in a study performed with brain metastases Singh et al observed that overexpression of miR-21 is capable to increase cell proliferation and sphere formation of their cells, while the influence on migration was not significant [40]. Moreover, modulated miR-21 activity failed to affect the migratory abilities of prostate cells [41]. Nonetheless, our data add confirmation for an oncogenic function of miR-21 in osteosarcoma.

The big challenge in treatment of osteosarcoma patients is resistance to the standard chemotherapy combining high-doses of methotrexate, doxorubicin, and cisplatin. Multiple and complex genetic and epigenetic factors are involved in modulating the sensitivity of osteosarcoma to the used compounds [42]. Thus, we aimed to elucidate the impact of miR-21 on mechanisms conferring resistance to these drugs. In our studies, we found that miR-21 activity had no influence on the sensitivity of osteosarcoma-derived cells towards doxorubicin and methotrexate. To our knowledge, this is the first approach investigating the effectiveness of a methotrexate treatment in dependency of miR-21 activity. Concerning sensitivity of tumor cells towards doxorubicin, the earlier reported studies suggest that the influence of miR-21 on the sensitivity of cells to this compound is dependent on the investigated tumor type. While for example in bladder cancer-derived cells [43] and in a glioblastoma-derived cell line [44] miR-21 expression decreased sensitivity of the cells towards doxorubicin, expression of a sponge in breast cancer derived cells had—like in our experiments with osteosarcoma-derived cells—no obvious influence [45]. In case of cisplatin treatment, miR-21 expression was shown to confer resistance to cisplatin in lung and gastric cancer [46] while in prostate cancer it had no effect [41]. In osteosarcoma, our presented data demonstrate that cells expressing a miR-21 sponge were more resistant towards cisplatin application. Corroborating, overexpressing miR-21 in the MG63 osteosarcoma cell line sensitized the cells to cisplatin treatment. This is in contrast to an earlier paper showing that miR-21 is associated with an increase in resistance towards cisplatin by using oligonucleotides inhibiting and mimicking miR-21 to modulate its activity [47]. This contradictory result of Ziyan et al could be a consequence of the application of oligonucleotides that might also influence miR-21-3p levels. In a very interesting study, Pink et al demonstrated that in ovarian cells cisplatin resistance is mediated by the miR-21-3p strand while the miR-21-5p strand is causing an opposite effect by increasing the sensitivity of their cells towards cisplatin [48].

Additionally we show that an increase of miR-21 expression as achieved by introducing a primiR-21 expressing vector into MG63 cells influenced the expression of the well-known miR-21 target Spry2 [16] and Spry1 [15] protein negatively. In contrast to data showing the importance of PDCD4 as a functional relevant target of miR-21 in breast cancer cells [18] as well as in colon cancer [19], in the osteosarcoma-derived cell line MG63 we failed to observe an influence of augmented miR-21 expression on PDCD4. Furthermore, PTEN levels were not influenced in MG63 cells.

Subsequently we observed that expression of Spry2 rescues the observed phenotypes induced by miR-21 as cell proliferation and chemo-sensitivity towards cisplatin in the presence of Spry2 were unaffected by the expression of primiR-21.

In conclusion, our observations indicate that the miR-21 regulatory network plays a role in tumorigenesis of osteosarcoma. Its expression facilitates cell proliferation and decreases cellular sensitivity towards cisplatin. Both effects can be rescued by Spry2, a target protein downregulated by increased miR-21 levels.
